# Microbiota and Inflammatory Markers: A Review of Their Interplay, Clinical Implications, and Metabolic Disorders

**DOI:** 10.3390/ijms26041773

**Published:** 2025-02-19

**Authors:** Emiliano Peña-Durán, Jesús Jonathan García-Galindo, Luis Daniel López-Murillo, Alfredo Huerta-Huerta, Luis Ricardo Balleza-Alejandri, Alberto Beltrán-Ramírez, Elsa Janneth Anaya-Ambriz, Daniel Osmar Suárez-Rico

**Affiliations:** 1 Licenciatura en Médico Cirujano y Partero, Centro Universitario de Ciencias de la Salud (CUCS), Universidad de Guadalajara, Guadalajara 44340, Mexico; 2 Departamento de Fisiología, Centro Universitario de Ciencias de la Salud (CUCS), Universidad de Guadalajara, Calle Sierra Mojada 950, Independencia Oriente, Guadalajara 44340, Mexico; 3 Departamento Académico Aparatos y Sistemas II, Decanato de Ciencias de la Salud, Universidad Autónoma de Guadalajara, Zapopan 44670, Mexico; 4 Departamento Académico Aparatos y Sistemas I, Decanato de Ciencias de la Salud, Universidad Autónoma de Guadalajara, Zapopan 44670, Mexico; 5 Hospital Medica de la Ciudad, Santa Catalina, Calle. Pablo Valdez 719, La Perla, Guadalajara 44360, Mexico; 6 Doctorado en Farmacología, Centro Universitario de Ciencias de la Salud, Universidad de Guadalajara, Guadalajara 44340, Mexico; 7 Departamento de Ciencias de la Salud, Centro Universitario de los Valles, Universidad de Guadalajara, Ameca 46708, Mexico; 8 Departamento de Farmacobiología, Centro Universitario de Ciencias Exactas e Ingenierías (CUCEI), Universidad de Guadalajara, Guadalajara 44430, Mexico

**Keywords:** microbiota, inflammation, biomarkers, probiotics, chronic diseases, dysbiosis, insulin resistance

## Abstract

The human microbiota, a complex ecosystem of microorganisms, plays a pivotal role in regulating host immunity and metabolism. This review investigates the interplay between microbiota and inflammatory markers, emphasizing their impact on metabolic and autoimmune disorders. Key inflammatory biomarkers, such as C-reactive protein (CRP), interleukin-6 (IL-6), lipopolysaccharides (LPS), zonulin (ZO-1), and netrin-1 (Ntn1), are discussed in the context of intestinal barrier integrity and chronic inflammation. Dysbiosis, characterized by alterations in microbial composition and function, directly modulates the levels and activity of these biomarkers, exacerbating inflammatory responses and compromising epithelial barriers. The disruption of microbiota is further correlated with increased intestinal permeability and chronic inflammation, serving as a precursor to conditions like type 2 diabetes (T2D), obesity, and non-alcoholic fatty liver disease. Additionally, this review **examines** therapeutic strategies, including probiotics and prebiotics, designed to restore microbial balance, mitigate inflammation, and enhance metabolic homeostasis. Emerging evidence positions microbiota-targeted interventions as critical components in the advancement of precision medicine, offering promising avenues for diagnosing and treating inflammatory and metabolic disorders.

## 1. Introduction

The gut microbiota is a diverse and dynamic community of microorganisms that plays an essential role in maintaining host health. It contributes significantly to immune system regulation, defense against infections, and the modulation of various physiological processes. [1,2]. The composition of microbiota can influence inflammation, a common factor in chronic diseases such as type 2 diabetes (T2D) and metabolic syndrome [3,4]. Probiotics, defined as live microorganisms administered in adequate amounts, can modulate the gut microbiota and, consequently, inflammatory biomarkers [5,6].

## 2. Microbiota and Its Main Components

The human microbiota, an extensive community of microorganisms inhabiting the body, comprises approximately 30–400 × 10^12^ cells predominantly located on epithelial surfaces, such as the skin and digestive tract [7,8]. Advances in next-generation sequencing technologies have revealed microbial populations in organs and tissues previously considered sterile, including the lungs, prostate, bladder, breast, liver, and pancreas [9]. These microorganisms span multiple taxonomic groups, predominantly bacteria, but also include viruses, protozoa, fungi, and archaea [10].

**Figure 1:**
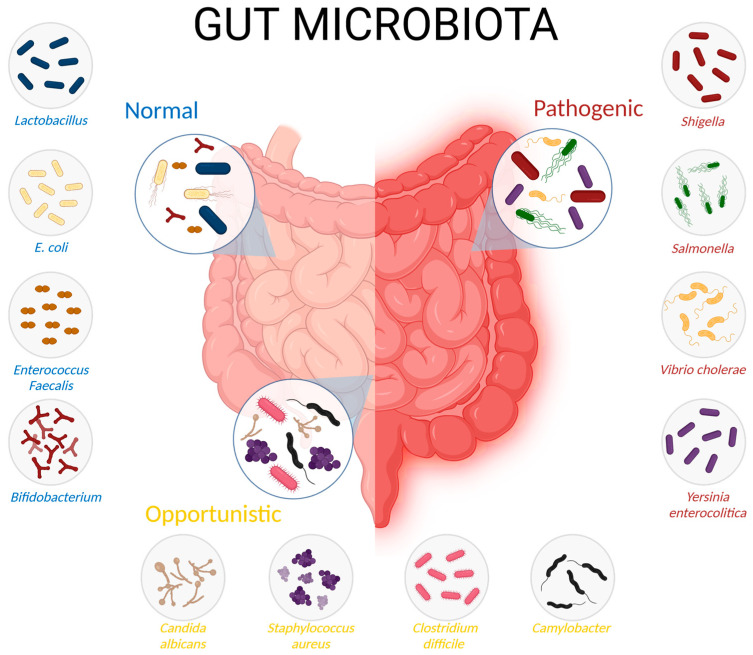
Gut Microbiota: Classification and Impact on Metabolic and Inflammatory Health. Classification of gut microbiota into three categories based on its impact on metabolic and inflammatory health: *normal microbiota* (essential for metabolic and defense functions, such as *Lactobacillus* and *Bifidobacterium*), *opportunistic microbiota* (can cause inflammation under dysbiosis conditions, such as *Candida albicans* and *Clostridium difficile*), and *pathogenic microbiota* (associated with systemic inflammation and metabolic disorders, such as *Salmonella* and *Yersinia enterocolitica*). Maintaining this balance is critical to preventing metabolic and inflammatory diseases.

The symbiotic relationship between humans and their microbiota is highly dynamic, adapting to specific niches and habitats throughout the body. These microorganisms coexist harmoniously and contribute to various physiological processes, functioning as integral components of the body from conception to death (Figure 1, Table 1) [7,10]. The microbiome produces a vast array of small molecules through de novo biosynthesis and host substrate modification, profoundly influencing mammalian physiology in both health and disease [11,12]. Notably, the collective genome of gut microbes alone surpasses the human genome by more than 100-fold [13].

Given this immense genetic potential, microbiota is implicated in nearly all physiological processes. It plays a pivotal role in transforming various compounds through biotransformation, aiding in detoxification, and facilitating the digestion of dietary lipids. The resulting metabolites can modulate local tissue responses and systemic immunity and metabolism in the host [7,14].

**Table 1:** Main microorganisms isolated in healthy patients [11,13,15,16,17,18,19,20,21,22,23,24,25,26].

Gastrointestinal Tract	
Bacteria	*Anaerofustis*, *Anaerostipes*, *Escherichia coli*, *Bacteroidetes*, *Firmicutes*, *Verrucomicrobia*, *Actinobacteria*, *Akkermansia muciniphila*, *Bacteroides coprophilus*, *Barnesiellaceae*, *Clostridiales*, *Faecalibacterium prausnitzii*, *Lachnospira*, *Rumminococcaceae*, *Christensenellaceae*, *Eubacterium dolichum*
Viruses	*Group 936 lactococcal phages*, *Picovirinae*, *Spounavirinae*, *Gokushovirinae*, *Acanthocystis turfacea chlorella virus 1*, *Marseilleviridae* and *Mimiviridae*
Fungi	*Candida* spp., *Cladosporium*, *Aureobasidium*, an unidentified *Saccharomycetales*, *Aspergillus*, and *Saccharomyces cerevisiae*, *in less proportion Malassezia and Epicoccum*
Lungs	
Bacteria	*Staphylococcus* spp., *Veillonella parvula*, *Fusobacterium nucleatum*, *Prevotella melaninogenica*, *Streptococcus parasanguinis*
Fungi	*Candida* spp.
Vagina	
Bacteria	Primarily *Lactobacilli* are present, *L. gasseri*, *L. iners*, and *L. crispatus*, *L. jensenii*, *L. plantarum*, *L. rhamnosus*
Fungi	*Candida* sp. is dominated by *C. albicans*, *Pichia kudriavzevii* and *C. parapsilosis*
Skin	
Bacteria	*Cutibacterium acnes*, *Staphylococcus epidermidis*, *Staphylococcus aureus*, *Enterococcus*, *Planococcaceae*
Fungi	*Malassezia* spp., *Aspergillus*, *Epicoccum*, and *Phoma*
Viruses	*Papillomaviridae*, *Polyomaviridae* and *Circoviridae.*
Acarides	*Demodex* spp.
Urinary tract	
Bacteria	*Gardnerella*, *Prevotella*, *Actinobaculum*, *Aerococcus*, *Corynebacterium*, *Gardnerella*, and *Streptococcus*
Eye conjunctiva	
Bacteria	*Staphylococcus aureus*, *Propionibacterium acnes* and *Peptostreptococcus* spp., *Propionibacterium* spp., *Bradyrhizobium* spp., *Brevundimonas* spp., *Aquabacterium* spp., *Sphingomonas* spp., *Simonsiella* spp., and *Methylobacterium* spp.
Fungi	*Fusarium solani*, *Cladosporium sphaerospermum*, *Acremonium implicatum*, *Candida albicans*, and *Aspergillus fumigatus*

## 3. Intestinal Microbiota and Inflammatory Biomarkers

The intestine plays a crucial role in regulating the immune system and protecting against pathogens, thereby having major effects on metabolic health. Disruption of the intestinal barrier, commonly referred to as “leaky gut”, is associated with chronic inflammation, a key factor in the development of diabetes. Therefore, identifying and understanding the inflammatory biomarkers linked to this process is essential for better addressing the immunological and metabolic underpinnings of this disease [27].

Various inflammatory biomarkers, such as IL-6, interleukin-10 (IL-10), hs-CRP, and lipopolysaccharide (LPS), are critical in the assessment of chronic inflammation associated with intestinal dysbiosis. These markers are essential for understanding how alterations in the intestinal microbiota and immune activation may contribute to the development of metabolic diseases such as diabetes. Specifically, IL-6 and hs-CRP are known for their pro-inflammatory functions, while IL-10 acts as an anti-inflammatory regulator. On the other hand, LPS, a component of gram-negative bacteria, has been implicated in immune system activation and the enhancement of systemic inflammation. These biomarkers may indicate a persistent inflammatory state that predisposes individuals to metabolic and autoimmune diseases, including forms of diabetes [27].

### 3.1. Interleukin-6 (IL-6)

IL-6 is a key cytokine in the regulation of the immune response and has been identified as an important regulator in a variety of pathological processes. Discovered in 1986, it is characterized by its pleiotropic activity, meaning it can exert different effects depending on the biological context. It is known for its ability to induce both acute and chronic inflammation, and its signaling occurs mainly through three distinct modes: classical signaling, trans-signaling, and cluster signaling. In classical signaling, IL-6 binds to the membrane-bound receptor (mIL-6R), while, in trans-signaling, IL-6 binds to the soluble form of this receptor (sIL-6R), which facilitates its activation in neighboring cells. This mode of signaling has been associated with a variety of inflammatory disorders, including autoimmune and metabolic diseases such as T2D [28]. Cluster signaling, also known as trans-presentation, involves IL-6 being presented by antigen-presenting cells (APCs) that express IL-6R directly to target immune cells, such as T cells, via cell-to-cell contact. This localized signaling is particularly relevant in tissue-specific immune responses [29]. IL-6 trans-signaling is crucial in intestinal dysbiosis, where alterations in the microbiota favor an increase in the levels of this cytokine, exacerbating systemic inflammation and contributing to insulin resistance [30]. IL-6 acts as a marker of chronic inflammation in T2D, and its elevation is linked to dysbiosis, reinforcing the need to better understand its role and potential strategies to modulate its signaling in the treatment of this disease [28,31].

Several clinical characteristics have been identified as factors that may influence IL-6 levels. These include weight gain, increased body mass index (BMI), dietary habits, and elevation of blood glucose concentrations [30]. Recent evidence has shown that drugs used to treat T2D, such as metformin, have a positive impact on the modulation of the intestinal microbiota, reducing IL-6 levels. This effect is partly mediated by bacterial species such as *Akkermansia muciniphila*, which are part of the intestinal microbiota. The reduction of IL-6 in peripheral blood has been associated with improvements in cognitive function in aged mice, highlighting the impact of this cytokine in both metabolic and cognitive diseases [32]. Additionally, it has been observed that IL-6 levels correlate positively with various bacterial species from the phylum Proteobacteria [33]. This inflammatory marker has been widely studied in metabolic diseases such as obesity, insulin resistance, and T2D. Elevated IL-6 concentrations have been shown to disrupt adipose tissue function and interfere with insulin action, a key hormone in the metabolism of various tissues, particularly muscle [34]. These metabolic disorders are associated with low-grade chronic inflammation [35]. In particular, the increase in cytokines, such as IL-6 in adipose tissue, can elevate the risk of developing insulin resistance, a key precursor to T2D. This phenomenon interferes with insulin recognition, leading to hyperinsulinemia and lipid accumulation in other tissues, such as the liver and adipose tissue, exacerbating disease progression [36].

### 3.2. Bacterial Lipopolysaccharide (LPS)

LPS is one of the inflammatory markers that has been studied in metabolic diseases, specifically obesity, insulin resistance, and T2D. This marker tends to increase in individuals with a high-fat diet, which is common among those with metabolic risk [34]

LPS is part of the outer membrane of Gram-negative bacteria, and its elevated peripheral levels, known as endotoxemia indicate changes in the intestinal microbiota, as well as clear damage to the intestinal epithelium, which functions as a barrier to prevent bacterial translocation. Endotoxemia has been positively correlated with inflammation and the development of metabolic alterations [37,38]. Recent evidence indicates that LPS plays a pivotal role in metabolic inflammation by activating the TLR4/NF-κB pathway, which induces the release of pro-inflammatory cytokines such as TNF-α, IL-6, and IL-1β. These cytokines contribute to systemic inflammation and impair insulin signaling by interfering with IRS-1 phosphorylation and the PI3K/AKT/GSK3β pathway, thereby promoting metabolic dysfunction. The persistent elevation of LPS is largely driven by diet-induced alterations in gut microbiota composition, where an overrepresentation of Proteobacteria has been correlated with increased LPS levels in both the liver and colon. These microbiota changes, coupled with intestinal barrier dysfunction, enhance LPS translocation into the bloodstream, sustaining chronic inflammation and insulin resistance [39]

In various metabolic contexts associated with pro-inflammatory processes, such as obesity, insulin resistance, and T2D, it has been identified that increases in cytokines, changes in the intestinal microbiota, as well as damage to the intestinal epithelium, bacterial translocation, and metabolic endotoxemia with elevated LPS, can affect insulin signaling in different tissues through an inflammatory process involving key kinases such as IKKβ, JNK, PKC, and JAK/STAT, all of which play crucial roles in promoting insulin resistance. These inflammatory kinases promote insulin resistance by negatively regulating key components of the insulin signaling cascade, such as the insulin receptor and insulin receptor substrate (IRS). Additionally, they induce the expression of cytokine signaling suppressors (SOCS), which inhibit insulin receptor signaling, thereby impairing normal insulin action in cells [40].

Among these kinases, JNK is activated by endoplasmic reticulum (ER) stress via IRE1α, leading to the phosphorylation of IRS-1 at Ser307, which disrupts insulin signaling and promotes the production of TNF-α and IL-6, intensifying the inflammatory response. Similarly, IKKβ/NF-κB is activated by TNF-α, which promotes the degradation of IκB, allowing NF-κB to translocate into the nucleus and induce the transcription of pro-inflammatory cytokines. Meanwhile, PKC is involved in metabolic inflammation by impairing GLUT4 translocation, reducing glucose uptake in muscle and adipose tissue, which further exacerbates insulin resistance. Additionally, the JAK/STAT pathway, activated by IL-6, contributes to insulin resistance by inducing the expression of SOCS proteins, which inhibit insulin receptor signaling, impairing normal insulin function [41].

The evaluation of LPS levels has been used as a marker of inflammation in individuals with T2D due to its clear association with common processes in this disease, such as obesity (increased BMI), glycemic dysregulation—characterized by abnormalities in blood sugar stability and reflected in elevated HbA1c levels—and inflammation (elevated IL-6 and hs-CRP) [42]. Although CRP remains a widely used marker of systemic inflammation, recent evidence suggests that specific lipidomic signatures, such as increased lactosyl ceramide (d18:1/16:0) and decreased phosphatidylcholine (18:0p/22:6), may offer more precise predictions of inflammatory conditions, including pediatric inflammatory bowel disease, and could reflect shifts in the gut microbiota [43].

### 3.3. Creactive Protein (CRP)

CRP is an acute-phase glycoprotein primarily produced in the liver in response to inflammatory stimuli, particularly IL-6. Its plasma concentration increases significantly during both acute and chronic inflammatory processes, making it a key biomarker in various pathologies, including T2D [44]. CRP has become established as a clinical marker of inflammation due to its ability to reflect the degree of immune system activation and its association with disease progression. In patients with chronic kidney disease (CKD), a common complication of T2D, elevated CRP levels have been shown to inversely correlate with glomerular filtration rate, suggesting its utility for monitoring renal function. Furthermore, CRP is involved in cardiovascular pathogenesis, a prevalent risk in T2D patients, mediated through the modulation of cell adhesion molecule expression and nitric oxide biosynthesis, thus reinforcing its relevance as a risk indicator in associated diseases [45,46,47,48]

In T2D, the increase in CRP reflects the low-grade chronic inflammation characteristic of this metabolic disease [49]. Several studies have shown that the elevation of this marker is closely correlated with glycemic dysregulation, as well as with alterations in the intestinal microbiota and damage to the intestinal epithelium, processes that promote the increase in serum lipopolysaccharides [50]. Both glycemic imbalance and changes in the intestinal microbiota, along with bacteremia, evidenced by the increase in the LPS levels in the bloodstream, have been identified as key inflammatory factors in the progression of T2D [51]. These mechanisms not only reflect the ongoing inflammatory state but have also been established as biomarkers contributing to the evaluation and monitoring of this metabolic disease, highlighting its evolution and associated complications [52].

### 3.4. Markers of Intestinal Epithelium Damage: ZO-1 and Ntn-1

The intestinal barrier is a complex system that protects the body from pathogens and regulates nutrient passage. It is formed by the interaction of bile, gastric acids, microbiota, and epithelial cells. The mucus layer, rich in mucins and antimicrobial peptides, prevents bacterial adhesion and promotes interaction with the intestinal microbiota. The epithelium, composed of different cell types, utilizes tight junctions to regulate permeability and maintain intestinal homeostasis. Proteins such as Zonulin (ZO-1), a component of tight junctions, and Ntn-1, involved in the regulation of intestinal development and function, are essential for the stability of this barrier [27,53,54]

Netrin-1, discovered in the 1990s, regulates key biological processes such as cell migration and axonal guidance. Through interactions with receptors such as DCC and Unc5b, it modulates cell adhesion, apoptosis, and proliferation. Additionally, it influences inflammation, tumorigenesis, epithelial repair, and angiogenesis, playing diverse roles depending on the cellular and tissue context [55]. Ntn-1, which is upregulated in the intestinal epithelium of patients with inflammatory bowel diseases (IBD), could be associated with intestinal epithelial damage by modulating leukocyte migration without directly affecting the epithelial barrier. Its assessment may serve as a biomarker not only for inflammation but also for tissue damage, as demonstrated by its protective effect in experimental colitis models. This mechanism could have implications in diabetes, where intestinal inflammation and epithelial barrier dysfunction play a crucial role in the development of metabolic complications [56,57,58].

ZO-1, discovered as the first tight junction protein, is essential for intestinal barrier function. Its structure includes specialized domains such as PDZ, SH3, and ABR, which enable its interaction with various proteins, such as claudins, occluding, and F-actin. ZO-1 organizes and regulates tight junctions and is involved in signal transduction and epithelial repair. Its dysfunction increases intestinal permeability, which is common in conditions with low-grade chronic inflammation, making it a risk biomarker. Although its inactivation in epithelia is not lethal, it causes an increase in macromolecular permeability, highlighting its key role in maintaining the epithelial barrier. Moreover, ZO-1 participates in essential processes such as Wnt/β-catenin signaling and epithelial repair [53,59].

## 4. Inflammatory Biomarkers and Insulin Resistance

Inflammatory biomarkers are biological indicators that reflect both normal states and pathological processes. Each of these biomarkers is present at different stages during the inflammatory response and exhibits varying degrees of sensitivity and specificity.

The most prominent biomarkers during chronic basal inflammation include high-sensitivity C-reactive protein (hs-CRP), while interleukin-6 (IL-6) is particularly useful in acute inflammation. Tumor necrosis factor-alpha (TNF-α) is one of the first cytokines released by macrophages and immune cells in response to acute stimuli. However, in chronic conditions such as obesity, insulin resistance, or autoimmune diseases, TNF-α can remain persistently elevated, albeit at lower levels compared to acute inflammation [1,60].

**Figure 2:**
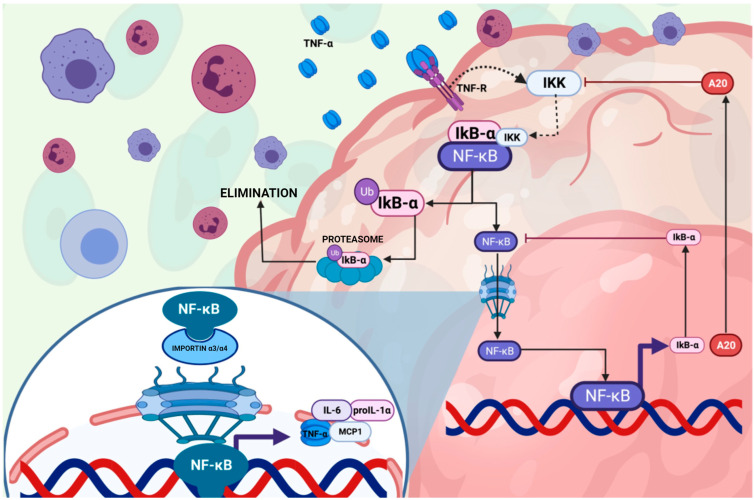
The NF-κB pathway activation by tumor necrosis factor α (TNF-α) and its impact on the regulation of inflammation at the cellular level. Tumor necrosis factor-alpha (TNF-α) through the NF-κB pathway and its impact on inflammatory regulation at the cellular level. The following key events are described: Production of TNF-α: Immune cells such as macrophages and lymphocytes release TNF-α in response to inflammatory stimuli. Receptor Interaction: TNF-α binds to the TNF-R receptor located in the plasma membrane, triggering an intracellular signaling cascade. IKK Complex Activation: TNF-α receptor binding recruits and activates the IKK (IκB kinase) complex, responsible for phosphorylating IκB-α, an inhibitory protein associated with the NF-κB transcription factor. IκB-α Degradation: Phosphorylation of IκB-α leads to its ubiquitination and subsequent degradation in the proteasome, releasing NF-κB. Nuclear Translocation of NF-κB: Once released, the NF-κB complex translocates to the nucleus, where it regulates the transcription of proinflammatory genes. Proinflammatory Gene Expression: NF-κB induces the expression of cytokines such as IL-6, IL-1β, and chemokines like MCP-1, which amplify the inflammatory response. Negative Regulation by A20: The diagram includes the role of A20 as a negative regulator of this pathway, inhibiting IKK complex activity to limit inflammatory signaling. Create by BioRender.com.

Moreover, TNF-α plays a pathological role by (1) Inhibiting insulin signaling in muscle and adipose tissues, thereby contributing to insulin resistance; (2) Promoting a low-grade inflammatory state through continuous activation of macrophages in adipose tissue; and (3) Stimulating the release of other chronic cytokines, perpetuating the inflammatory cycle [2,3,61,62] (Figure 2).

Insulin resistance is defined as a decreased activity of the insulin pathway signaling in peripheral tissues, primarily skeletal muscle, liver, and adipose tissue, resulting in part from physical inactivity, a hypercaloric diet, obesity, and molecular irregularities such as inefficient expression or activation of insulin receptors [63].

Key proteins involved in insulin signaling, including the insulin receptor (IR), insulin receptor substrate (IRS), and phosphatidylinositol 3-kinase (PI3K), play critical roles in the development of insulin resistance. Additionally, polymorphisms in IRS genes and serine/threonine phosphorylation of IRS contribute significantly to impaired insulin signaling [64].

Currently, two pathophysiological phenomena are described to explain the onset of insulin resistance. The first affects insulin receptor substrate (IRS) activity, a decrease in which leads to reduced phosphorylation of downstream proteins and further decreased GLUT4 translocation to the cell membrane, thereby diminishing insulin activity. The second mechanism is related to lipotoxicity caused by excess free fatty acids (FFAs) production. FFA accumulation in hepatic tissue results in insulin resistance, characterized by reduced glucose consumption by hepatocytes. This process subsequently induces elevated serum insulin concentration, generating new FFA molecules and completing the cycle. This mechanism, known as the “twin cycle”, posits that it is self-stimulating production of FFAs that impairs pancreatic beta-cell function that is responsible for insulin resistance, especially in genetically susceptible individuals [65,66].

While these changes occur, a common feature in both mechanisms is the inflammatory state. This inflammation can manifest as chronic basal inflammation, typically observed in individuals with obesity, or as a hyperacute inflammatory response. Both states can be evaluated using different inflammatory markers. Inflammatory markers associated with hyperacute states linked to insulin resistance include IL-6, while hs-CRP is associated with chronic basal inflammation.

**Figure 3:**
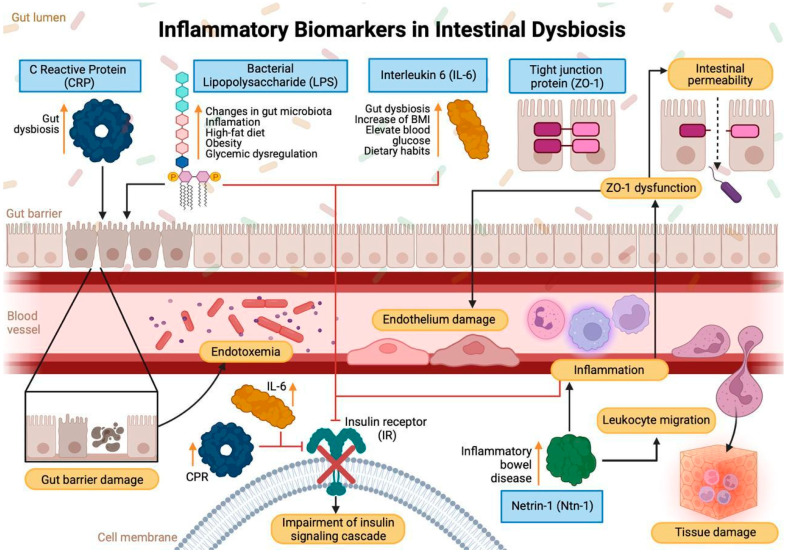
Inflammatory biomarkers in intestinal dysbiosis. This diagram illustrates the primary factors that disrupt intestinal barrier homeostasis and their systemic repercussions. High-fat diets, obesity, and glycemic dysregulation contribute to dysbiosis, increasing the release of bacterial lipopolysaccharides (LPS). LPS activates Toll-like receptors and the NF-κB pathway, promoting the production of pro-inflammatory cytokines such as interleukin-6 (IL-6), which in turn stimulates hepatic synthesis of C-reactive protein (CRP). These inflammatory responses impair tight junction proteins, including ZO-1, leading to increased intestinal permeability and facilitating bacterial translocation (endotoxemia). Systemically, endotoxemia induces endothelial damage, leukocyte infiltration, and impaired insulin signaling, exacerbating chronic inflammation and metabolic dysfunction. Conversely, Netrin-1 exerts a regulatory effect by limiting immune cell infiltration and preserving the integrity of the intestinal barrier. The imbalance between pro-inflammatory mediators and protective mechanisms highlights the critical role of maintaining barrier function to preserve systemic homeostasis.

Emerging biomarkers associated with insulin resistance include netrin 1 (Ntn-1), a cell migration guidance protein comprising 640 amino acids. Ntn-1 is involved in neuronal navigation, immune cell migration, and cellular survival. It demonstrates a plasma concentration of 100–150 ng/dL in healthy subjects. Its activity is strictly related to its receptor binding, Unc5b, which, upon activation, generates phosphorylation of the peroxisome proliferator-activated receptor-γ (PPAR-γ). This transcription factor increases adiponectin expression. Furthermore, Ntn1 inhibits the activity of the nuclear factor kappa B (NF-κB) transcription factor, reducing the expression of pro-inflammatory cytokines dependent on this factor (Figure 3) [67,68,69]. Recent studies have identified a correlation between elevated serum levels of Ntn-1 and patients with insulin resistance and T2D [70].

## 5. Clinical Implications

The commensal gut microbiota and its metabolites can significantly attenuate low-grade systemic inflammation by indirectly influencing intestinal barrier integrity [71].

Lipopolysaccharides (LPS), a component of the outer cell wall of Gram-negative bacteria, are well-known bacterial endotoxins. Elevated plasma levels of LPS associated with chronic inflammation are referred to as metabolic endotoxemia. This condition is characterized by serum LPS levels that are two-three times higher than normal and is associated with a variety of diseases, including obesity, T2D, non-alcoholic fatty liver disease (NAFLD), chronic kidney disease, and cardiovascular disease [34]. Endotoxemia activates a signaling cascade in which LPS binds to toll like receptor 4 (TLR4) complex, activating the Toll-like receptor 4 (TLR4) and stimulating NF-κB to produce inflammatory cytokines such as tumor necrosis factor-α (TNF-α) and interleukin-1β (IL-1β) [72]. Such activation of inflammatory signaling increases intestinal permeability, exacerbating chronic low-grade inflammation.

Short-chain fatty acids (SCFAs) produced by the gut microbiota also interact with intestinal hormone signaling to influence feeding behavior. SCFAs bind to specific G-protein coupled receptors (GPRs), including GPR41 and GPR43, promoting the release of peptide YY (PYY), which enters circulation and interacts with the hypothalamus to reduce total food intake [73]. Butyrate, a specific SCFA, reduces energy expenditure by increasing plasma levels of glucagon-like peptide-1 (GLP-1), glucose-dependent insulinotropic peptide (GIP), and PYY, all of which have key implications in managing T2D due to their effects on glucose metabolism, insulin sensitivity, and appetite regulation [74]. In a randomized clinical trial, propionate, another SCFA, was also shown to stimulate the release of PYY and GLP-1. Moreover, it upregulates genes involved in intestinal gluconeogenesis by binding to GPR41, thereby reducing adiposity [75].

Dietary interventions, particularly those involving the Mediterranean diet, have been shown to be effective in improving depressive symptoms. Observational studies and rand omized controlled trials suggest that whole-diet interventions, such as the Mediterranean diet, can significantly reduce depressive symptoms in clinical samples [76] The Mediterranean diet, which emphasizes the consumption of fruits, vegetables, whole grains, nuts, and olive oil, has been associated with a lower risk of depression and cognitive decline [77], “Furthermore, the PREDIMED study demonstrated that the Mediterranean diet, supplemented with virgin olive oil or nuts, significantly reduces inflammatory biomarkers such as C-reactive protein and IL-6, supporting its role in mitigating systemic inflammation and lowering cardiovascular risk [78].

Beyond dietary interventions, more direct approaches such as fecal microbiota transplantation (FMT) have demonstrated significant potential in modulating gut microbiota composition and influencing disease progression. For instance, a randomized controlled trial revealed that FMT can halt the progression of new-onset type 1 diabetes by preserving residual beta-cell function, highlighting its potential in managing autoimmune and metabolic disorders [79,80]. However, the outcomes of FMT are highly variable depending on the donor microbiota composition. Additionally, there is an inherent risk of transferring pathogens or inducing unintended dysbiosis, particularly in immunocompromised patients, which underscores the need for stringent donor screening and patient selection protocols [81].

Gut microbiota dysbiosis has also been linked to obesity-associated NAFLD [82]. Patients with NAFLD often exhibit bacterial overgrowth and reduced intestinal barrier integrity. Worsening course of NAFLD has been associated with increased populations of Bacteroides, while liver fibrosis correlates with higher levels of Ruminococcus [83]. The gut microbiota is indirectly involved in hepatic triglyceride deposition through interactions with fasting-induced adipose factor (FIAF). FIAF, an inhibitor of lipoprotein lipase secreted by enterocytes, is suppressed by gut microbiota [84]. FIAF also activates carbohydrate response element-binding protein and sterol regulatory element-binding protein 1, ultimately upregulating triglyceride production and accumulation in the liver [84].

The evidence presented underscores the central role of probiotics and their metabolites in modulating the gut microbiota and influencing various clinical conditions, including metabolic, inflammatory, and hepatic diseases. Through complex mechanisms involving intestinal barrier regulation, the production of SCFAs such as butyrate, and the modulation of key metabolic pathways, probiotics not only enhance metabolic homeostasis but also mitigate chronic inflammatory processes that exacerbate pathologies such as T2D, NAFLD, and obesity. These findings highlight the importance of incorporating microbiota-targeted interventions into clinical management, laying the groundwork for innovative therapeutic strategies that promote holistic and personalized health care.

## 6. The Symbiotic Role of Probiotics and Prebiotics in Modulating Microbiota and Inflammatory Markers

Scientific evidence increasingly highlights the interconnected nature of disease pathophysiology, with a shared proinflammatory pattern emerging as a central mechanism in various conditions. Among the key factors involved, imbalances in the human microbiota stand out due to their profound impact on multiple systems and metabolic pathways. The gastrointestinal tract, as a critical hub of this interaction, plays a central role in what is now recognized as the “Microbiota-Gut Axis”. This concept underscores the bidirectional communication between microbiota alterations and the progression of systemic inflammatory disorders, including metabolic syndrome and autoimmune diseases [85].

Probiotics, defined as live, non-pathogenic microorganisms, have gained attention for their potential therapeutic benefits [86]. They can be bacteria, fungi, or yeasts, which—when administered in adequate amounts (at least 10^6^ colony-forming units per gram)—provide health benefits by improving physicochemical balance and participating in metabolism, sometimes serving as a therapeutic alternative [87,88].

Disruption of the host–microbiota balance, also called dysbiosis, stems from multiple factors such as pathogenic microorganisms, the deterioration and loss of beneficial microbiota, age, diet, the use of antimicrobials, and underlying medical conditions [89].

Such disruption and its subsequent harm are counteracted by probiotics, given that they stimulate the immune system and confer competitive resistance against infectious pathogens [88], as well as preserve and enhance the endogenous microbiota through a “barrier effect” in various axes, via different mechanisms of action [87,89].

Among the described properties of probiotics are resistance to acidic pH, tolerance to bile salts and pancreatic fluids, and the capacity to adhere to and invade intestinal epithelial cells [87,88].

Among the most commonly administered microorganisms and strains for supplementation are *Lactobacillus* spp. and *Bifidobacterium* spp., which are isolated from the human intestine and have the greatest evidence base regarding their taxonomic derivatives [87], as well as *Streptococcus* spp., *Enterococcus* spp., *Saccharomyces boulardii*, and *Bacillus* spp., among others [85,86].

These probiotics are beneficially attributed with mechanisms such as antagonism of pathogenic organisms; competition for luminal space (spatial disposition theory); nutrient synthesis as an energy source; maintenance and preservation of cytoprotective integrity in the intestinal and gastric mucosa; regulation of intestinal motility through neuroendocrine stimulation and reciprocal communication between the central and enteric nervous systems; hypolipidemic effects facilitated by bile acid deconjugation and by binding or co-precipitation of FFAs; anticancer, antimutagenic, and antiallergic properties; production and regulation of reactive oxygen species for epithelial repair and restitution; the synthesis of antimicrobial agents, organic acids, and bacteriocins; among others, such as protective effects against primary and secondary osteoporosis by increasing bone density and offering protection against estrogen deficiency [87].

Moreover, multiple organisms demonstrate a bidirectional influence at the central nervous system level, modulating the hypothalamic–pituitary–adrenal axis, tryptophan metabolism, and their capacity to synthesize various neurotransmitters and produce metabolites such as short-chain fatty acids (acetate, butyrate, lactate, and propionate), hypothesized to be involved in the microbiota–gut–brain axis [90].

This explains how certain strains exert effects on the innate immune system through the stimulation of Toll-like receptors, favoring macrophage-mediated phagocytosis. At the intestinal level, there is activation of CD4+ and CD8+ lymphocytes, secretion of immunoglobulin A, and a multifaceted suppression of the inflammatory response mediated by Th1 and inflammatory cytokines such as IL-12 and TNF-alpha, among others [87].

Regular and adequate probiotic intake promotes intestinal colonization in the population without directly impairing the functional microbiota of the host. This interaction is reflected at the mucosal level and in GALT (gut-associated lymphoid tissue), modulating the function of antigen-presenting cells and intestinal epithelial cells. Consequently, a key response involving dendritic cells, macrophages, and intraperitoneal lymphocytes is observed. This effect has shown inhibitory capacity on the NF-κβ cascade and its possible connection with the MAPK signaling, ultimately reducing inflammatory cytokines and their mediators, such as IL-8. An increase in the cytotoxicity of NK cells and macrophage phagocytosis has also been demonstrated, resulting in inhibitory and regulatory effects on the M1 subtype macrophages (proinflammatory; mediated by TNF, IL-1, IL-6, IL-8, and IL-12) and stimulating the M2 subtype macrophages (anti-inflammatory; regulated by cytokines, including IL-10, IL-1Ra, and transforming growth factor beta) [88].

Certain *Bifidobacterium* or *Lactobacilli* strains contain cell-wall components such as lipoteichoic acid (a TLR2 ligand), capable of stimulating nitric oxide synthase in macrophages, thus increasing the capacity of phagocytosis receptors such as FcγRIII and TLRs—key steps in initiating the early immune response and differentiating specific subtypes of CD4+ cells (Th1, Th2, or Th17) [88].

In the intestine, this results in an indirect effect on the intestinal biofilm and its function as barriers, increased mucin production, induction of antimicrobial peptides, and the production of heat shock proteins, leading to increased IgA and decreased T-cell proliferation. Consequently, the pathogenic influence of proinflammatory and immunomodulatory cells is modulated [88].

Certain probiotics, such as *Lactobacillus plantarum*, have demonstrated the ability to inhibit TNF-α production by reducing the degradation of IκB-α and IκB-β. Additionally, pretreatment with this strain inhibits the phosphorylation of ERK, JNK, and p38 MAPKs kinases in THP-1 monocytic cells, indicating modulation of endotoxin signaling pathways and tolerance. This effect has been studied with pathogens such as *Staphylococcus aureus* and *Shigella flexneri*, enhancing an anti-inflammatory modulation of their pathogenicity [88].

Other specific *Lactobacillus* strains have demonstrated, in immunoallergic disorders, bidirectional effects characterized by increases in cytokines and transcriptional regulators (Th1 type, IFN-γ, IL-10, IL-12, TGF-β, FoxP3), immunoglobulins (IgG, IgG1, IgG2a, IgA), and cells (NK, Treg), as well as reductions in inflammatory mediators (Th2 type, IL-4, IL-5, IL-6, IL-13, TNF-α), globulins (IgE and IgG4), CD86 expression, and neutrophil infiltration. Clinically, these strains have improved overall and emotional quality of life, negatively impacting the risk of atopy, eczema, rhinitis, episode duration, mucosal hypersecretion, and limitations on daily activities [91].

Multiple studies have elucidated the therapeutic potential of probiotics for immune modulation and inflammatory suppression, through their capacity for cellular regulation and differentiation, improvement in intestinal permeability, and reduction in the passage of LPS and other inflammatory mediators into the circulation [92].

The influence of the microbiomes and the effect of dysbiosis on the microbiota–brain axis plays a pivotal role in the development of neuropathology such as Alzheimer’s disease (lowering malondialdehyde, high-sensitivity CRP, and other neuromodulators) [93,94], dementia, Parkinson’s disease (GLP-1 pathway promotion, PPAR-γ activation, increased sphingolipid metabolism, decreased lipid peroxidation, and reductions in IL-6, TNF-α, peripheral and central ROS, along with improvements in motor and nonmotor functions) [95], and attention-deficit/hyperactivity disorder [90].

Likewise, the impact of the microbiota–gut–brain axis is related to depression, with evidence that the combined and/or supplementary use of specific strains, called “psychobiotics,” mitigates overall symptoms, improves clinical test outcomes, and regulates expression of immunomodulators and neurotransmitters such as norepinephrine, corticosterone, GABA, and cortisol [96].

A notable example is the use of *Lactobacillus plantarum JYLP-326*, which suggests a potentially effective index in the presence of transient predisposing factors such as insomnia, anxiety, or depression and their relationship with the microbiota [97].

Similar effects have been analyzed in the oral cavity, suggesting the therapeutic potential of supplementation in synergy with antimicrobial substances use, enhancing prophylactic effects and reducing pathogen counts, clinically improving preexisting conditions, and achieving a significant reduction in pathology associated markers such as matrix metalloproteinase-8 (MMP-8) and IL-6 [98].

Recent literature has not reported significant discoveries on the relationship between prebiotics, symbiotics, and inflammatory biomarkers since 2019. However, systematic reviews and meta-analyses published from that year have integrated and analyzed data primarily generated before this period [99,100,101]. These analyses suggest a potential trend towards a reduction in inflammatory biomarkers following the use of prebiotics and symbiotics; however, these effects have not consistently reached statistical significance, limiting definitive conclusions about their effectiveness.

Notably, much of the research conducted since 2019 has shifted focus toward the central nervous system, exploring the role of prebiotics and symbiotics in conditions such as Parkinson’s disease and ADHD, and multiple sclerosis [102,103,104,105,106]. For example, the study ‘Targeting gut microbiota: new therapeutic opportunities in multiple sclerosis” explores the potential of modulating gut microbiota, including the use of prebiotics and symbiotics, as an innovative therapeutic approach for managing multiple sclerosis. The research emphasizes how gut–brain axis modulation can influence inflammatory pathways, offering new perspectives in the treatment of neuroinflammatory diseases.

**Figure 4:**
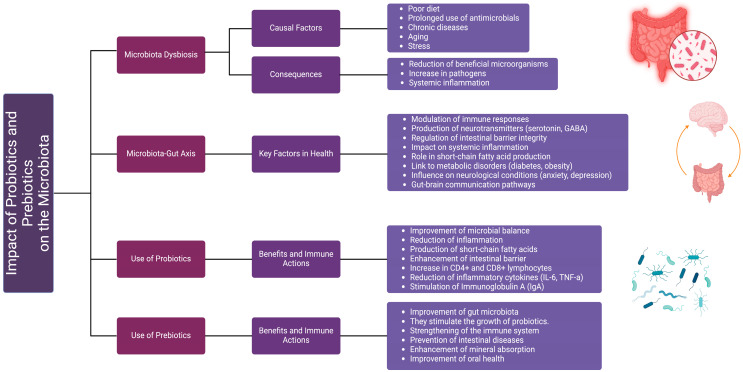
Impact of probiotics and prebiotics on microbiota. The diagram illustrates the effects of probiotics and prebiotics on gut microbiota and their role in maintaining health. It highlights the causal factors and consequences of microbiota dysbiosis, the key functions of the microbiota–gut axis in health, and the specific benefits of probiotic and prebiotic use. Probiotics contribute to microbial balance, inflammation reduction, and immune modulation, while prebiotics stimulate the growth of beneficial bacteria, enhance mineral absorption, and support intestinal health. The figure also emphasizes the interplay between gut microbiota and systemic processes, including immune responses and neurological conditions.

While these studies provide valuable insights into the potential neuromodulator’s effects of these interventions, they leave a significant gap in understanding their impact on inflammatory biomarkers, particularly in non-neurological contexts. Nevertheless, promising studies are currently underway to address these limitations and generate new evidence. For instance, Chamani and collaborators are conducting a randomized controlled trial protocol titled “Investigating the effect of symbiotic supplementation on inflammatory indices in critically ill septic children: a protocol study for randomized control trial” (Figure 4) [107]. This study seeks to assess the effects of symbiotic supplementation on inflammatory markers in critically ill septic children. Beyond evaluating the efficacy of these interventions, this study seeks to contribute to the development of personalized approaches for managing inflammation in vulnerable pediatric populations.

Such investigations are crucial for further exploring specific mechanisms, identifying more sensitive biomarkers, and establishing standardized protocols to maximize the therapeutic potential of these interventions. The current gap in generating robust and significant data represents an opportunity for future research to deepen understanding and expand the clinical application of these strategies.

## 7. The Role of Pathogenic Microorganisms in Inflammation: Implications for Chronic Diseases

The human microbiota plays an essential role in maintaining intestinal homeostasis. However, when pathogenic microorganisms are present, this balance can be disrupted, triggering local and systemic inflammatory responses. Among the most studied pathogens are *Escherichia coli*, *Clostridium difficile*, and *Helicobacter pylori*. These microorganisms affect not only the intestinal environment but also have broader implications for metabolic and autoimmune diseases, primarily through the activation of the immune system [108,109].

One of the key mechanisms behind this inflammation involves LPS, structural components of Gram-negative bacteria like *Escherichia coli*. LPS interact with TLR4 on immune cells, initiating a cascade that leads to the production of pro-inflammatory cytokines such as TNF-α and IL-6 [110]. This process not only drives local inflammation but can also result in a chronic, low-grade inflammatory state, often referred to as metabolic endotoxemia, which has been linked to obesity and insulin resistance [111].

Other pathogens, such as *Clostridium difficile*, produce toxins that directly damage intestinal epithelial cells, causing severe inflammation and weakening the intestinal barrier. This damage allows microorganisms to enter the bloodstream, further intensifying systemic inflammation. In a similar way, *Helicobacter pylori* contribute to chronic gastric inflammation by releasing virulence factors and enzymes that stimulate cytokine production and cause ongoing cellular damage [112,113].

The presence of these pathogens also disrupts the composition of the microbiota, creating an imbalance that perpetuates inflammation. Beneficial bacteria such as *Akkermansia muciniphila* and *Faecalibacterium prausnitzii* decrease in number, while harmful microorganisms flourish. This imbalance exacerbates immune and metabolic dysfunction, contributing to diseases such as Crohn’s disease, ulcerative colitis, and various metabolic and cardiovascular conditions [3,114,115].

**Figure 5:**
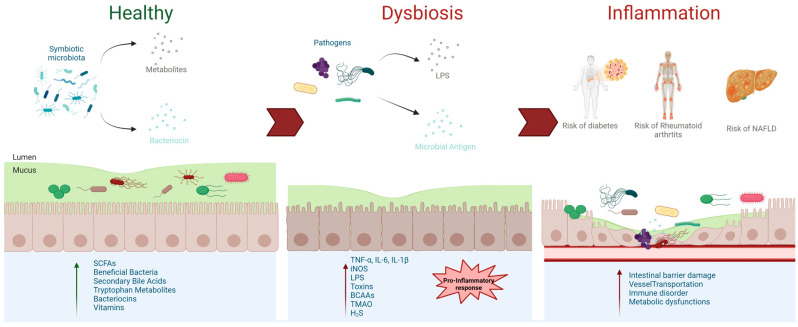
Progression from Healthy Microbiota to Dysbiosis and Inflammation. The figure illustrates the transition from a symbiotic gut microbiota to a state of dysbiosis and its impact on systemic inflammation. In a healthy microbiota, beneficial bacteria produce essential metabolites such as short-chain fatty acids (SCFAs), secondary bile acids, and bacteriocins, which support intestinal barrier integrity, immune regulation, and metabolic health. Dysbiosis disrupts this balance, leading to increased production of harmful substances such as lipopolysaccharides (LPS), microbial antigens, and pro-inflammatory cytokines (e.g., TNF-α, IL-6, IL-1β). This pro-inflammatory response contributes to intestinal barrier damage, immune dysregulation, angiogenesis, and systemic inflammation, increasing the risk of metabolic disorders such as diabetes, rheumatoid arthritis, and non-alcoholic fatty liver disease (NAFLD). Abbreviations: BCAA, branched-chain amino acids; TMAO, trimethylamine N-oxide; iNOS, inducible nitric oxide synthase.

Understanding how these pathogens interact with the immune system and microbiota is crucial for developing effective interventions. Probiotics, prebiotics, and targeted antibiotics may help to restore microbiota balance and reduce inflammation. The symbiotic gut microbiota plays a crucial role in maintaining overall health through various mechanisms. It integrates into the intestinal barrier, producing bacteriocins and beneficial metabolites such as short-chain fatty acids (SCFAs) and bile acids, which prevent pathogen colonization of the intestinal epithelium [116]. Additionally, it supports digestive metabolism and fosters the development and regulation of the immune system (Figure 5). Therapeutic approaches that aim to repair the intestinal barrier and regulate immune responses hold promise for preventing and managing chronic diseases associated with dysbiosis and inflammation.

Pathogenic microorganisms clearly play a significant role in promoting inflammation, with far-reaching effects on both intestinal and systemic health. By addressing the mechanisms through which these pathogens disrupt microbiota and immune balance, we can pave the way for innovative treatments that target the root causes of chronic inflammatory conditions.

## 8. Future Perspectives on Microbiota and Biomarkers

Growing evidence highlights microbiota as both a critical diagnostic indicator and a potential therapeutic target. Specific alterations in microbial composition have been linked to early stages of colorectal cancer (CRC), suggesting their potential utility as a disease biomarker [117,118]. Similar associations extend to pancreatic conditions [119] and neurodegenerative disorders like Parkinson’s disease, where LPS affects inflammation and intestinal barrier function [120]. Alongside LPS, markers such as IL-6 and ZO-1 underscore the microbiota’s pivotal role in regulating inflammation [121,122,123].

Advancements in therapeutic strategies include the application of probiotics, which have demonstrated antihypertensive and anti-inflammatory effects [124]. Moreover, exosomes derived from mesenchymal stem cells (MSCs) have shown promise in strengthening the intestinal barrier in colitis models [125]. Complementing these approaches, electrochemical biosensing devices now offer enhanced precision in monitoring microbial markers, facilitating timely detection of dysbiosis [126]. Although challenges remain regarding methodological standardization and establishing causality between microbial shifts and disease, current data suggest that microbiota modulation and monitoring will be integral to precision medicine [127,128].
